# Nomogram based on computed tomography images and clinical data for distinguishing between primary intestinal lymphoma and Crohn’s disease: a retrospective multicenter study

**DOI:** 10.3389/fmed.2023.1246861

**Published:** 2023-08-17

**Authors:** Mengjun Xiao, Jiahe Tan, Haiou Li, Chenyang Qiu, Yinchao Ma, Haiyan Wang

**Affiliations:** ^1^Department of Radiology, Shandong Provincial Hospital Affiliated to Shandong First Medical University, Jinan, Shandong, China; ^2^Computer Science Graduate Studies, University of California, Davis, Davis, CA, United States; ^3^Qilu Hospital, Cheeloo College of Medicine, Shandong University, Jinan, Shandong, China

**Keywords:** primary intestinal lymphoma, Crohn’s disease, nomogram, computer tomography, diagnosis

## Abstract

**Background:**

Differential diagnosis of primary intestinal lymphoma (PIL) and Crohn’s disease (CD) is a challenge in clinical diagnosis.

**Aims:**

To investigate the validity of the nomogram based on clinical and computed tomography (CT) features to identify PIL and CD.

**Methods:**

This study retrospectively analyzed laboratory parameters, demographic characteristics, clinical manifestations, and CT imaging features of PIL and CD patients from two centers. Univariate logistic analysis was performed for each variable, and laboratory parameter model, clinical model and imaging features model were developed separately. Finally, a nomogram was established. All models were evaluated using the area under the curve (AUC), accuracy, sensitivity, specificity, and decision curve analysis (DCA).

**Results:**

This study collected data from 121 patients (PIL = 69, CD = 52) from Center 1. Data from 43 patients (PIL = 24, CD = 19) were collected at Center 2 as an external validation cohort to validate the robustness of the model. Three models and a nomogram were developed to distinguish PIL from CD. Most models performed well from the external validation cohort. The nomogram showed the best performance with an AUC of 0.921 (95% CI: 0.838–1.000) and sensitivities, specificities, and accuracies of 0.945, 0.792, and 0.860, respectively.

**Conclusion:**

A nomogram combining clinical data and imaging features was constructed, which can effectively distinguish PIL from CD.

## 1. Introduction

The intestine is the most common site of extranodal lymphoma other than the stomach, with the ileum being the most common (1). Primary intestinal lymphoma (PIL) is very rare, accounting for less than 4% of all gastrointestinal malignancies, and most are non-Hodgkin’s lymphomas (2). The pathological biopsy is considered to be the gold standard for diagnosing PIL. However, there is a possibility of a negative biopsy due to the small size or superficiality of the specimen. In addition, the manifestations in clinical of PIL are not specific, so it is often confused with other intestinal diseases (3).

Crohn’s disease (CD) is an idiopathic inflammatory disease with a slow course, often alternating between relapses and remissions, influenced by genetic, immunological and environmental factors (4, 5). The diagnosis of CD relies not only on tissue biopsy but also on a combination of clinical signs, laboratory tests, and imaging (5). CD most commonly occurs in the terminal ileum and ileocecal region, which has similarities with PIL (6). In addition, there are overlapping aspects of PIL and CD in terms of clinical signs and imaging manifestations, which increases the difficulty of differential diagnosis between PIL and CD.

It is noteworthy that the treatment of PIL and CD is completely different. PIL is mainly treated with surgery or chemotherapy (7, 8). However, CD is usually treated with pharmacological treatment for induction and maintenance (5). Delayed diagnosis of PIL and CD may lead to poor prognosis and disease complications (9). Therefore, accurate and rapid diagnosis of PIL and CD can help in the selection of treatment options, which is a great challenge for clinicians.

Therefore, we retrospectively collected data on clinical features, laboratory parameters, and radiological characteristics of PIL and CD patients. The aim is to develop an effective and simple diagnostic model that would be helpful in differentiating PIL from CD.

## 2. Materials and Methods

### 2.1. Subjects

**Figure 1:**
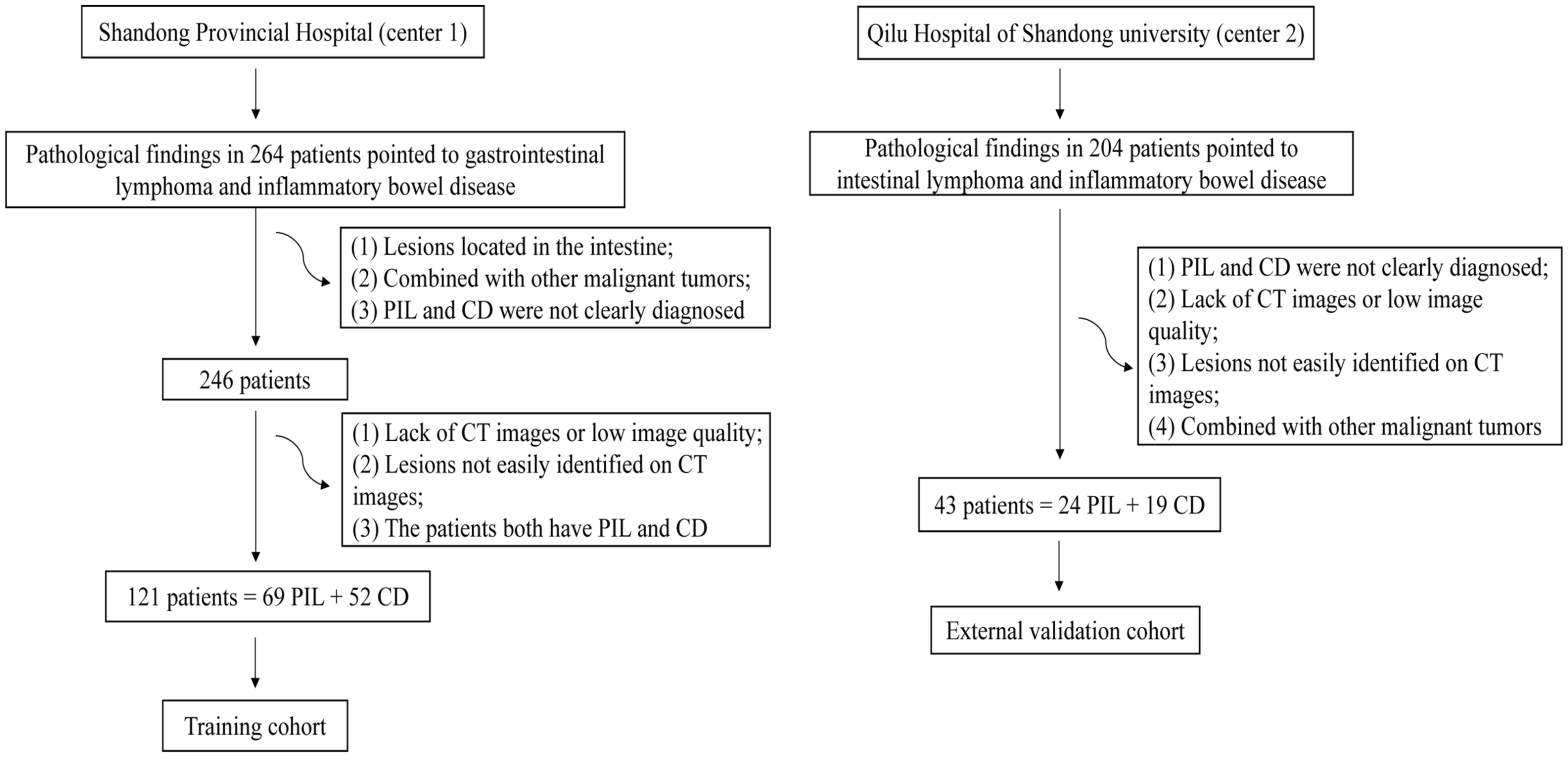
Flowchart of participant selection.

We searched medical records for 264 patients diagnosed with PIL and CD from January 2011 to December 2022 at the Shandong provincial hospital affiliated to Shandong First Medical University (Center 1). Finally, 121 patients who met the inclusion criteria (69 patients with histologically confirmed PIL and 52 patients with clinically diagnosed CD including biopsy pathology) were included. We collected 43 patients approved by Qilu Hospital of Shandong University (Center 2) from June 2015 to August 2022. Due to the retrospective nature of this study, the requirement of informed consent was waived. Figure 1 shows the flowchart of participant selection.

### 2.2. Methods

Patients with PIL were included according to the Dawson criteria (10). Patients with CD were included according to the following criteria: (1) clinical diagnosis of CD; (2) no previous intestinal surgical treatment. All the above patients underwent at least one computed tomography (CT) examination and pathological examination during hospitalization. All of the above patients were excluded according to the following criteria: (1) patients with both PIL and CD; (2) patients with other gastrointestinal malignancies; and (3) lack of the required medical imaging images. Basic clinical data were recorded and displayed. Non-enhanced, arterial-phase, and venous-phase CT images were collected from all patients.

### 2.3. Data collection

Clinical information about the patients including demographic characteristics, laboratory parameters, and clinical manifestations and imaging features was obtained from the electronic medical record.

Demographic parameters included gender and age of onset.

Laboratory data were recorded as follows: hemoglobin level, platelet count, albumin level, lymphocyte absolute value, neutrophil cell absolute value, eosinophil absolute value, and C-reactive protein (CRP) level.

Clinical manifestations include time from onset to diagnosis, abdominal pain, diarrhea, bloating, bloody stools, fever, increased frequency of stools, abdominal mass, tenesmus, and weight loss.

**Figure 2:**
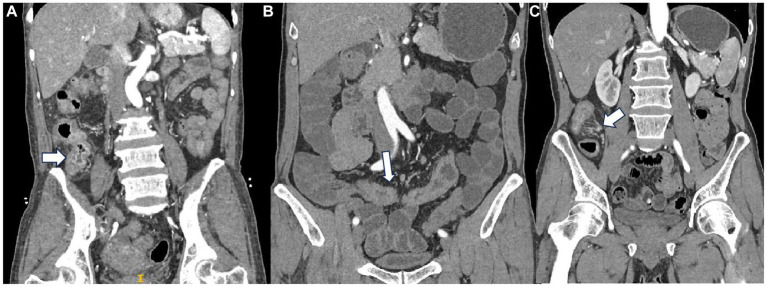
**(A)** Mucosal enhancement a patient with PIL. **(B)** Homogeneous enhancement in a patient with CD. **(C)** “Comb sign” in a patient with CD.

All included patients had undergone at least one CT examination and were evaluated by two experienced radiologists. The CT features include intestinal wall thickness, intestinal stenosis, aneurysmal dilatation, enlargement of the abdominal lymph nodes, the enhanced density of the peri-intestinal fat, “comb sign,” the degree and mode of enhancement after enhancement scan, and CT values in each phase (Figure 2). Consider dilatation or stenosis of the intestinal lumen as observed in at least two planes at the lesion.

In terms of enhancement methods, tumors with low or no enhancement areas within the tumor are considered to have heterogeneous enhancement. Layered enhancement is considered to be mucosal, relatively poor submucosal, and serosal enhancement (11, 12). The enhancement of the innermost layer of the intestinal wall is mucosal enhancement. For the level of enhancement, compared with plain CT, an increase of 10–30 HU in the CT value in enhanced CT images is defined as a mild enhancement. The increase in CT value of the lesion at a level of 30–50 HU is defined as a moderate enhancement. More than 50 HU is defined as a severe enhancement. Segmentation of CT images in plain, arterial, and venous phases was performed using ITK-SNAP (RRID:SCR_017341) (version 4.0, http://www.itksnap.org) (13). The region of interest (ROI) was delineated by the physician with a multi-layer manual outline of the lesion area, excluding the intestinal lumen and vessels.

### 2.4. Statistical analysis

Several scales were designed to analyze information on patients’ demographic, clinical, laboratory, and imaging characteristics. Patients in the PIL and CD group were analyzed using IBM SPSS Statistics (RRID:SCR_019096) (version 25.0; SPSS, Inc., Chicago, IL, United States) and GraphPad Prism (RRID:SCR_002798) (version 9.0; GraphPad, San Diego, CA). Continuous variables that were normally distributed were expressed as mean ± SD, otherwise median (upper and lower quartiles) was used. Categorical variables were expressed as frequencies and percentage values. All statistical tests were two-sided. *p* < 0.05 was considered a statistically significant difference. Missing values were all less than 20%, and missing values were filled using multiple interpolations. Predictive mean matching (PMM) was chosen to interpolate the data 20 times, and then the interpolated results were split and summarized to generate the final dataset.

First, univariate logistic analysis was performed for each variable. Then, parameters with *p* < 0.05 and AUC ≥0.6 (rounded) were integrated, and the laboratory parameters model, clinical model combining demographic data with clinical symptoms, and imaging features model, respectively, were developed using R Project for Statistical Computing (RRID:SCR_001905) (version 4.2.3, https://www.r-project.org/). In addition, covariate diagnostics were performed for each model’s variables. Finally, the indicators with *p* < 0.05 in the three models were integrated and the nomogram was plotted.

In addition, receiver operating characteristic (ROC) curves were plotted to assess the discrimination. The DeLong test was used to compare the AUC between models. The Hosmer–Lemeshow goodness-of-fit test was used to determine the goodness-of-fit of the nomogram and calibration curves were plotted to assess the agreement between the predicted and actual results. Sensitivity, specificity, and accuracy were calculated to assess the performance of all models. Finally, clinical decision curves (DCA) were plotted to understand patient benefits. Patients from Center 2 served as an external validation set to demonstrate the robustness of the model. The characteristics of the two centers are shown in Table 1. Most of the characteristics of the two centers are not statistically different.

**Table 1:** Baseline characteristics of the training cohort and the external validation cohort.

Parameter	Training cohort (Center 1)		External validation cohort (Center 2)		*p*-value
	PIL	CD	PIL	CD	
Gender (male/female) (%)	50 (72.5)/19 (27.5)	30 (57.7)/22 (42.3)	13 (54.2)/11 (45.8)	12 (63.2)/7 (36.8)	0.349
Age of onset (year)	52.59 ± 19.08	44.96 ± 18.67	64.13 ± 14.27	47.68 ± 18.57	0.026^*^
Time from onset to diagnosis (month)	2.00 (1.00, 6.00)	12.00 (2.25, 36.00)	2.50 (1.00, 6.00)	6.00 (2.00, 96.00)	0.355
Abdominal pain (%)	60 (87.0)	45 (86.5)	21 (87.5)	18 (94.7)	0.500
Diarrhea (%)	7 (10.1)	28 (53.8)	2 (8.3)	1 (5.3)	0.007^*^
Bloating (%)	27 (39.1)	17 (32.7)	9 (37.5)	5 (26.3)	0.654
Bloody stool (%)	8 (11.6)	12 (23.1)	4 (16.7)	3 (15.8)	0.970
Increased frequency of stools (%)	5 (7.2)	32 (61.5)	2 (8.3)	6 (31.6)	0.131
Fever (%)	10 (14.5)	18 (34.6)	3 (12.5)	2 (10.5)	0.106
Abdominal mass (%)	17 (24.6)	1 (1.9)	6 (25.0)	0 (0.0)	0.883
Tenesmus (%)	4 (5.8)	6 (11.5)	0 (0.0)	0 (0.0)	0.052
Weight loss (%)	25 (36.2)	30 (57.7)	12 (50.0)	5 (26.3)	0.502
Hemoglobin (g/L)	119.64 ± 19.55	108.73 ± 25.24	107.83 ± 22.84	120.16 ± 16.15	0.673
Platelet (109/L)	294.00 (231.00, 344.50)	327.50 (264.25, 475.25)	226.00 (171.50, 295.50)	300.00 (257.00, 336.00)	0.013^*^
Lymphocyte absolute value (109/L)	1.44 (1.02, 1.94)	1.44 (1.08, 2.01)	1.07 (0.70, 1.24)	1.35 (1.12, 1.65)	0.006^*^
Neutrophil cell absolute value (109/L)	4.32 (3.18, 5.23)	4.99 (3.44, 7.25)	4.54 (3.33, 7.61)	4.36 (2.81, 5.22)	0.788
Eosinophil absolute value (109/L)	0.06 (0.03, 0.15)	0.07 (0.03, 0.15)	0.09 (0.02, 0.23)	0.07 (0.04, 0.16)	0.261
Albumin (g/L)	36.67 ± 5.25	34.18 ± 5.40	36.96 ± 5.74	39.35 ± 4.33	0.012^*^
C-reactive protein (mg/L)	17.14 (6.53, 35.62)	28.31 (6.56, 60.12)	25.08 (10.05, 27.69)	25.43 (9.00, 31.02)	0.960
Intestinal wall thickness (mm)	17.44 (13.76, 25.62)	9.74 (7.31, 12.35)	18.01 (11.75, 21.08)	10.25 (9.34, 14.79)	0.999
Intestinal stenosis (%)	40 (58.0)	46 (88.5)	14 (58.3)	17 (89.5)	0.899
Aneurysmal dilation (%)	28 (40.6)	4 (7.7)	12 (50.0)	1 (5.3)	0.633
Enlargement of the abdominal lymph nodes (%)	55 (79.7)	26 (50.0)	15 (62.5)	13 (68.4)	0.828
Enhanced density of the peri-intestinal fat (%)	28 (40.6)	38 (73.1)	16 (66.7)	14 (73.7)	0.082
Comb sign (%)	14 (20.3)	45 (86.5)	1 (4.2)	15 (78.9)	0.192
Mild enhancement	61 (88.4)	37 (71.2)	18 (75.0)	14 (73.7)	0.361
Moderate enhancement	7 (10.1)	14 (26.9)	6 (25.0)	4 (21.1)	0.396
Severe reinforcement	1 (1.4)	1 (1.9)	0 (0.0)	1 (5.3)	0.777
Homogeneous enhancement	54 (78.3)	11 (21.2)	18 (75.0)	4 (21.1)	0.773
Layered or mucosal enhancement	10 (14.5)	40 (76.9)	2 (8.3)	12 (63.2)	0.312
Non-enhanced phase CT value	40.24 ± 7.55	39.53 ± 6.09	38.94 ± 5.78	35.29 ± 5.53	0.029^*^
Arterial phase CT value	60.71 ± 12.44	65.74 ± 13.10	61.04 ± 11.55	58.90 ± 10.23	0.210
Intravenous phase CT value	68.41 ± 11.99	74.02 ± 12.71	70.67 ± 10.75	67.24 ± 10.62	0.440

^*^p < 0.05. PIL, primary intestinal lymphoma; CD, Crohn’s disease.

## 3. Results

### 3.1. Patients

A total of 121 patients from Center 1 (52 CD and 69 PIL) and 43 patients from Center 2 (19 CD and 24 PIL) were included in this study.

### 3.2. Demographic features

No significant difference was found between PIL and CD patients in terms of gender. However, the age of onset in patients with the PIL group was significantly higher than those in the CD group (52.59 ± 19.08 years vs. 44.96 ± 18.67 years, *p* < 0.05).

### 3.3. Clinical manifestations

In terms of clinical presentation, the time from onset to diagnosis was significantly longer in the CD group than in the PIL group [median time, 12.00 (2.25, 36.00) mo vs. 2.00 (1.00, 6.00) mo, *p* < 0.05]. The incidence of diarrhea, increased frequency of stools, fever, and weight loss was significantly higher in the CD than in PIL (*p* < 0.05). In contrast, the incidence of abdominal masses was significantly higher in the PIL group than in the CD (*p* < 0.05). One-way logistic regression analysis of demographic characteristics of primary intestinal lymphoma and Crohn’s disease are presented in Table 2.

**Table 2:** One-way logistic regression analysis of demographic characteristics of primary intestinal lymphoma and Crohn’s disease.

Parameter	Training cohort (Center 1)			
	PIL (*n* = 69)	CD (*n* = 52)	Logistic regression analysis	
			*p*-value	AUC
Gender (male/female) (%)	50 (72.5)/19 (27.5)	30 (57.7)/22 (42.3)	0.091	0.574
Age of onset (year)	52.59 ± 19.08	44.96 ± 18.67	0.032^*^	0.620^#^
Time from onset to diagnosis (month)	2.00 (1.00, 6.00)	12.00 (2.25, 36.00)	0.002^*^	0.768^#^
Abdominal pain (%)	60 (87.0)	45 (86.5)	0.946	0.502
Bloating (%)	27 (39.1)	17 (32.7)	0.467	0.591
Bloody stool (%)	8 (11.6)	12 (23.1)	0.098	0.557
Increased frequency of stools (%)	5 (7.2)	32 (61.5)	0.000^*^	0.771^#^
Abdominal mass (%)	17 (24.6)	1 (1.9)	0.007^*^	0.614^#^
Tenesmus (%)	4 (5.8)	6 (11.5)	0.265	0.529
Weight loss (%)	25 (36.2)	30 (57.7)	0.020^*^	0.607^#^

^*^p < 0.05; #AUC ≥ 0.6; AUC, area under the curve; PIL, primary intestinal lymphoma; CD, Crohn’s disease.

### 3.4. Laboratory parameters

Laboratory tests showed no significant difference in lymphocyte absolute value and eosinophil absolute value between the PIL group and CD group. CRP level, platelet count, and neutrophil cell absolute value were significantly higher in the CD group compared to the PIL group (*p* < 0.05). Albumin and hemoglobin levels were lower in the CD group compared to the PIL group. One-way logistic regression analysis of laboratory parameters of primary intestinal lymphoma and Crohn’s disease are listed in Table 3.

**Table 3:** One-way logistic regression analysis of laboratory parameters of primary intestinal lymphoma and Crohn’s disease.

Parameter	Training cohort (Center 1)			
	PIL (*n* = 69)	CD (*n* = 52)	Logistic regression analysis	
			*p*-value	AUC
Hemoglobin (g/L)	119.64 ± 19.55	108.73 ± 25.24	0.011^*^	0.632^#^
Platelet (10^9^/L)	294.00 (231.00, 344.50)	327.50 (264.25, 475.25)	0.013^*^	0.628^#^
Lymphocyte absolute value (10^9^/L)	1.44 (1.02, 1.94)	1.44 (1.08, 2.01)	0.868	0.529
Neutrophil cell absolute value (10^9^/L)	4.32 (3.18, 5.23)	4.99 (3.44, 7.25)	0.019^*^	0.602^#^
Eosinophil absolute value (10^9^/L)	0.06 (0.03, 0.15)	0.07 (0.03, 0.15)	0.416	0.512
Albumin (g/L)	36.67 ± 5.25	34.18 ± 5.40	0.014^*^	0.616^#^
C-reactive protein (mg/L)	17.14 (6.53, 35.62)	28.31 (6.56, 60.12)	0.013^*^	0.593

^*^p < 0.05; #AUC ≥ 0.6; AUC, area under the curve; PIL, primary intestinal lymphoma; CD, Crohn’s disease.

### 3.5. Computed tomography imaging features

CT examination showed that aneurysmal dilatation of the lesion area and enlarged abdominal lymph nodes were more common in the PIL group than in the CD group (*p* < 0.05). Patients with CD had significantly more intestinal stenosis, the enhanced density of the peri-intestinal fat, and “comb sign” at the lesion than patients with PIL (*p* < 0.05). The intestinal wall was thickened in both PIL and IBD patients, but significantly thicker in PIL patients than in CD patients [median time, 17.44 (13.76, 25.62) mm vs. 9.74 (7.31, 12.35) mm, *p* < 0.05]. On enhancement scans, PIL more often showed homogeneous, mild enhancement, whereas CD tended to have moderate, stratified, or mucosal enhancement (*p* < 0.05). In addition, the CT values of lesions in the arterial and venous phases were higher in patients with CD compared to PIL (*p* < 0.05). One-way logistic regression analysis of computed tomography imaging features of primary intestinal lymphoma and Crohn’s disease are listed in Table 4.

**Table 4:** One-way logistic regression analysis of computed tomography imaging features of primary intestinal lymphoma and Crohn’s disease.

Parameter	Training cohort (Center 1)			
	PIL (*n* = 69)	CD (*n* = 52)	Logistic regression analysis	
			*p*-value	AUC
Intestinal wall thickness (mm)	17.44 (13.76, 25.62)	9.74 (7.31, 12.35)	0.000^*^	0.887^#^
Aneurysmal dilation (%)	28 (40.6)	4 (7.7)	0.000^*^	0.664^#^
Comb sign (%)	14 (20.3)	45 (86.5)	0.000^*^	0.831^#^
Mild enhancement	61 (88.4)	37 (71.2)	0.020^*^	0.586
Moderate enhancement	7 (10.1)	14 (26.9)	0.020^*^	0.584
Severe reinforcement	1 (1.4)	1 (1.9)	0.840	0.502
Layered or mucosal enhancement	10 (14.5)	40 (76.9)	0.000^*^	0.812^#^
Non-enhanced phase CT value	40.24 ± 7.55	39.53 ± 6.09	0.575	0.534
Arterial phase CT value	60.71 ± 12.44	65.74 ± 13.10	0.037^*^	0.594
Intravenous phase CT value	68.41 ± 11.99	74.02 ± 12.71	0.018^*^	0.624^#^

^*^p < 0.05; #AUC ≥ 0.6; AUC, area under the curve; PIL, primary intestinal lymphoma; CD, Crohn’s disease.

### 3.6. Development of differentiation models of PIL with CD patients

Comparative analysis of laboratory parameters, clinical manifestations, and imaging features was performed to establish the best model with the best discriminatory ability.

**Figure 3:**
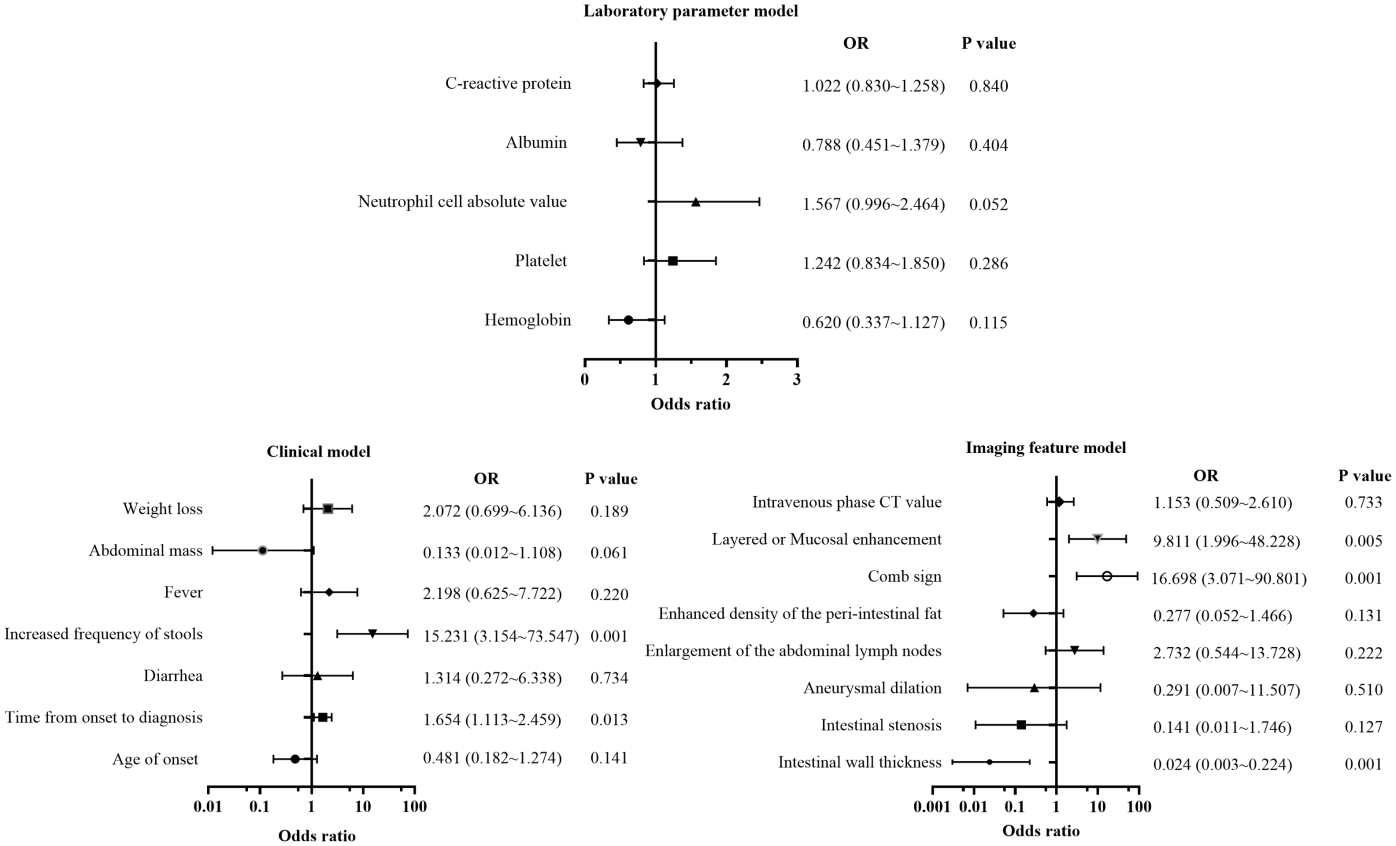
Forest plot of multivariate regression analysis based on laboratory parameters model, clinical model, and imaging features model.

**Figure 4:**
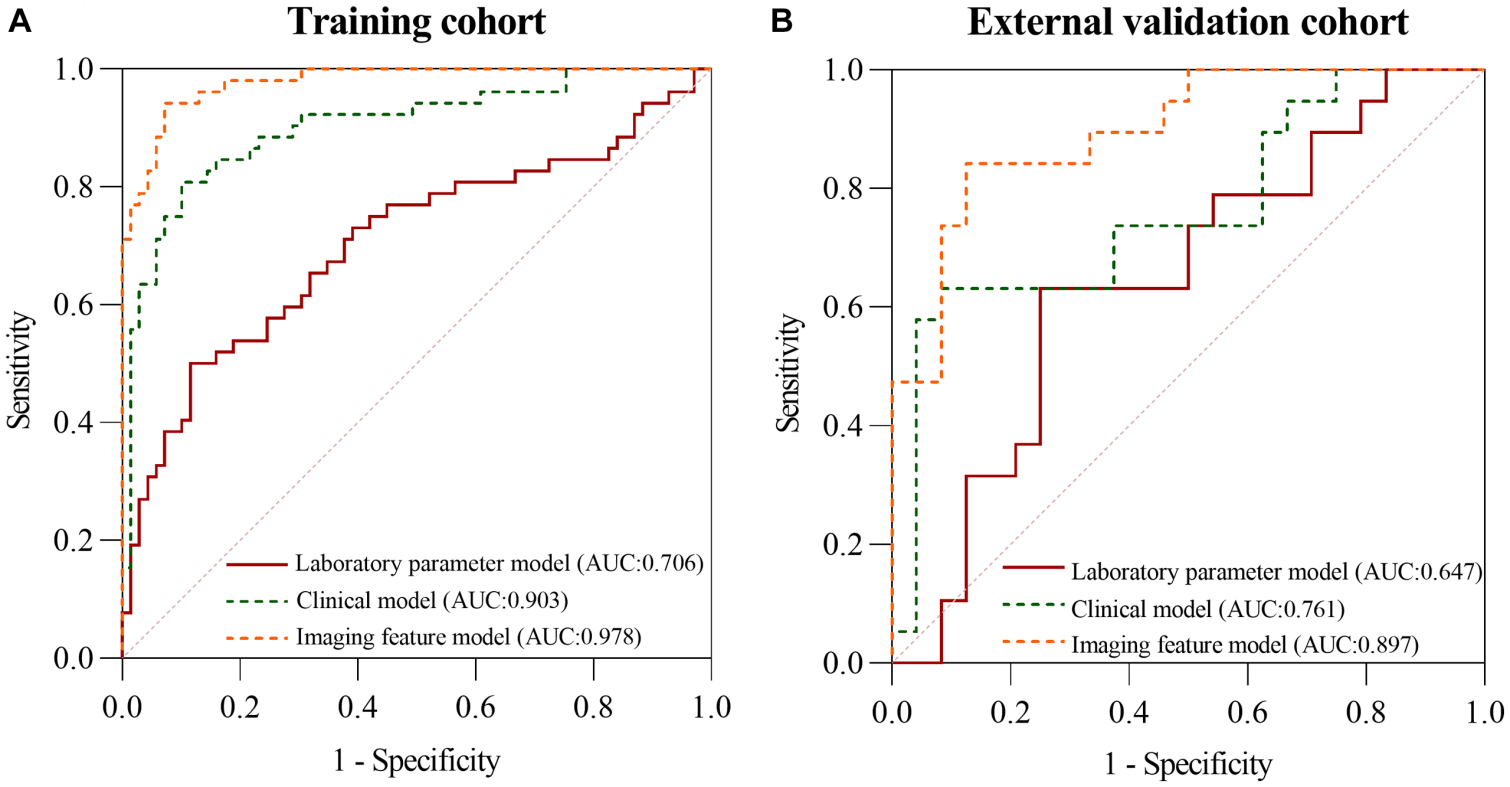
The ROC curves of the laboratory parameters model, clinical model, and imaging features model in the training cohort **(A)** and the external validation cohort **(B)**, respectively.

First, all indicators were analyzed separately by univariate logistic analysis, and those with *p* < 0.05 and AUC ≥0.6 in the univariate logistic analysis were included in multivariate logistic regression analysis. The laboratory parameters model, clinical model, and imaging features model were developed and covariate diagnoses were performed. Hemoglobin, albumin, and CRP levels as well as platelet counts and neutrophil cell absolute value were included in the laboratory parameters models. Age of onset, time from onset to diagnosis, diarrhea, increased frequency of stools, fever, abdominal mass, and weight loss were included in the clinical model. Imaging features model included intestinal wall thickness, intestinal stenosis, aneurysmal dilatation, enlargement of the abdominal lymph nodes, the enhanced density of the peri-intestinal fat, “comb sign” and layered or mucosal enhancement at the lesion, and venous phase CT values. In the training cohort and external validation cohort, the AUCs of the laboratory parameters model, clinical model, and imaging features model were 0.706 and 0.647; 0.903 and 0.761; and 0.978 and 0.897, respectively. Forest plots and ROC curves of the three models are shown in Figures 3, 4.

### 3.7. Development and evaluation of Nomogram

**Figure 5:**
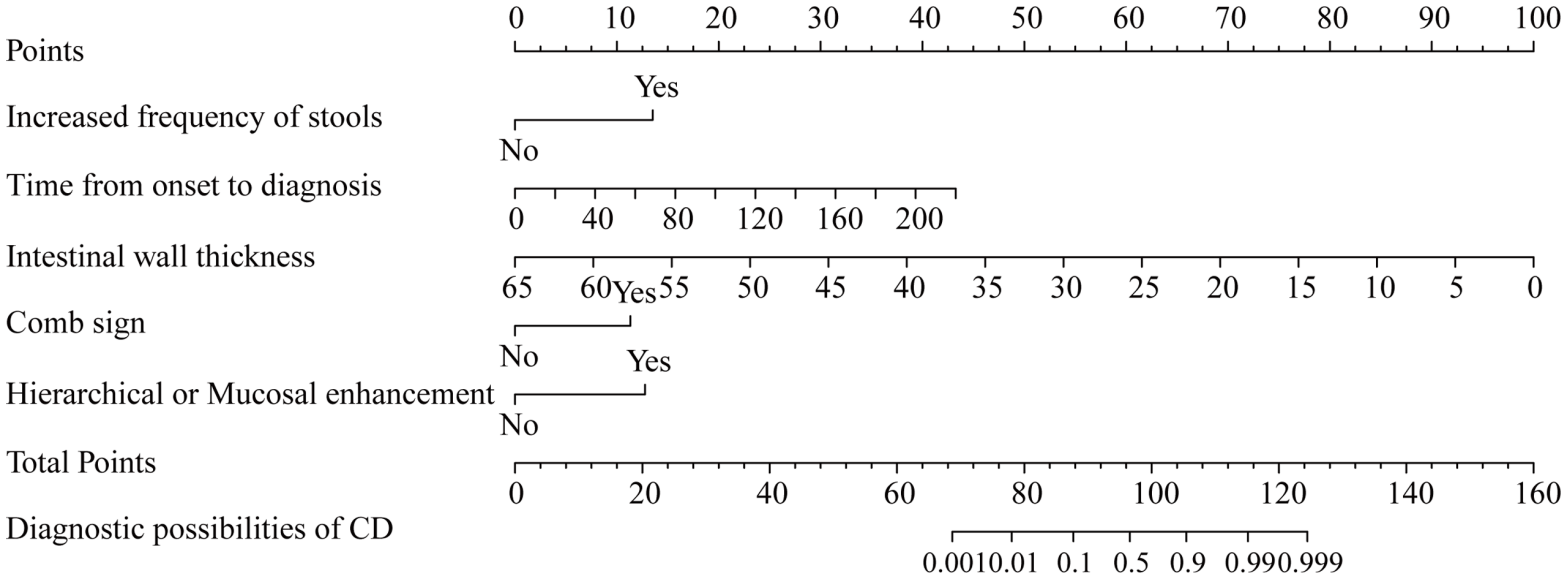
Nomogram based on clinical signs and imaging features.

The indicators with *p* < 0.05 in the above model were selected to build a nomogram, including time from onset to diagnosis, increased frequency of stools, intestinal wall thickness, and “comb sign” with layered or mucosal enhancement at the lesion. The nomogram was plotted in Figure 5. The scores of the nomogram were calculated as follows:

Nomogram=0.8475+3.3488×Increased frequency of stools+0.0487×Time from onset to diagnosis−0.3811×Intestinal wall thickness+2.8057×Combsign+3.1613×Layered or mucosal enhancement.

**Figure 6:**
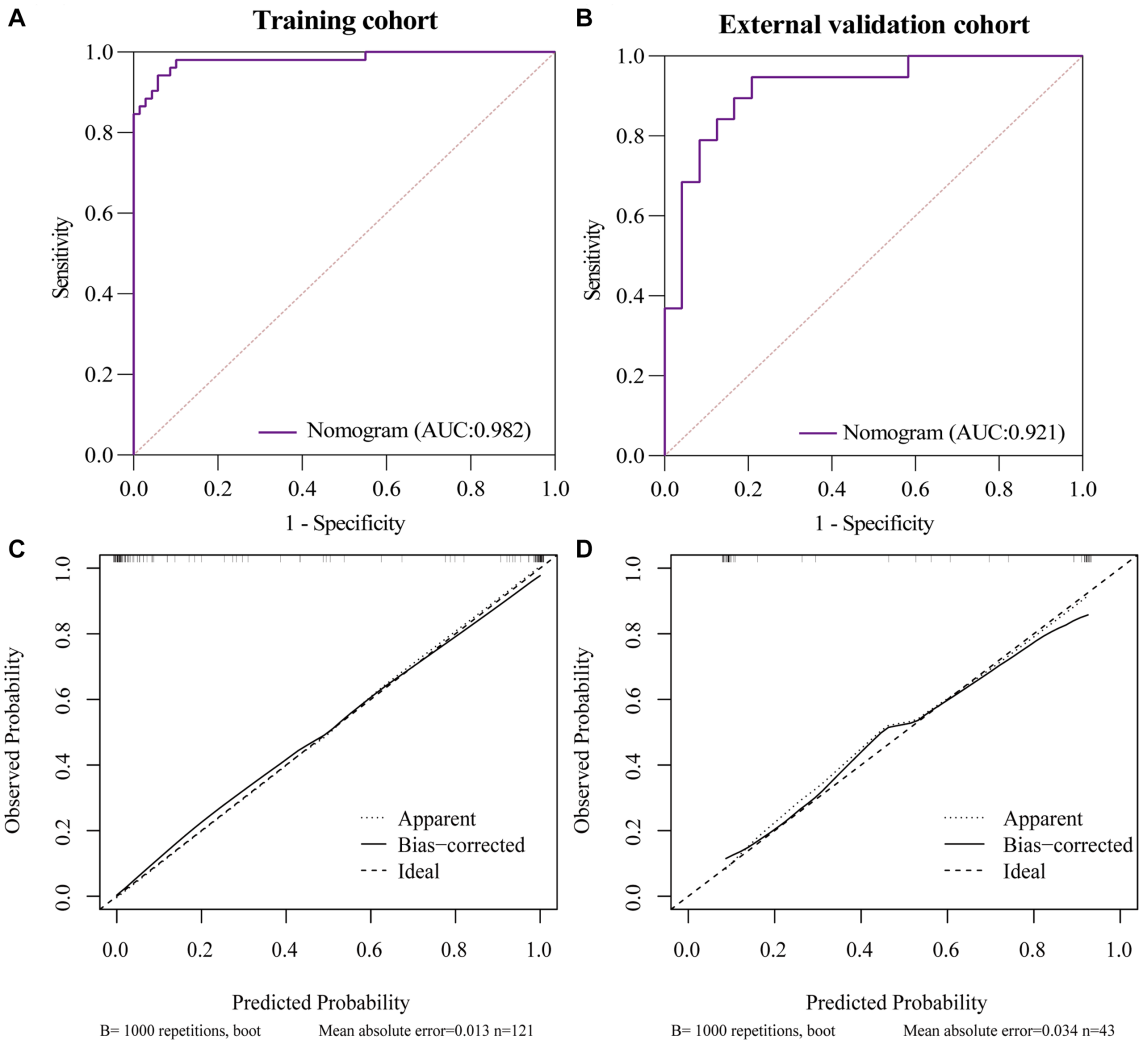
ROC and Calibration curves of Nomogram in training cohort and external validation cohort. **(A)** ROC curve of nomogram in the training cohort. **(B)** ROC curve of nomogram in the external validation cohort. **(C)** Calibration curve of nomogram in the training cohort. **(D)** Calibration curve of nomogram in the external validation cohort.

**Figure 7:**
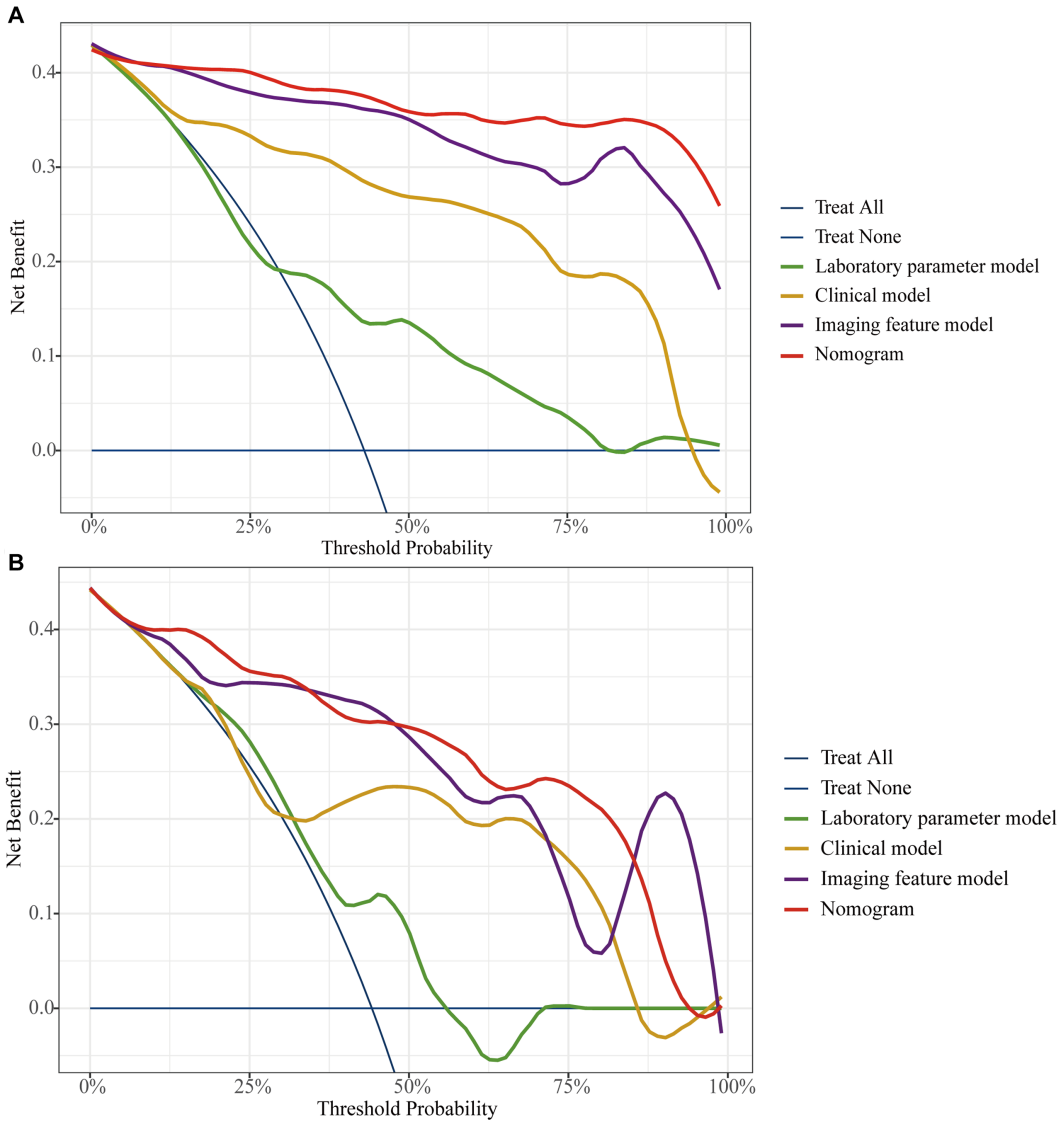
The decision curve analysis for all models in training cohort **(A)** and external validation cohort **(B)**.

The AUC of the nomogram was 0.982 and 0.921 for the training cohort and the external validation cohort, respectively. The DeLong test was used to compare the AUC between models. In the training cohort and external validation cohort, the nomogram had no statistical significance with the image feature model (*p* = 0.5527/0.8753) but had statistical significance with other models (*p* < 0.05). The nomogram fit well in the training cohort (*p* = 0.148) and the external validation cohort (*p* = 0.660) according to the Hosmer–Lemeshow goodness-of-fit test. The nomogram shows good agreement in the calibration curves of both cohorts (Figure 6). In addition, the decision curves for the laboratory parameters model, clinical model, imaging features model, and nomogram are shown in Figure 7. Nomogram showed the highest net benefit, followed by the imaging features model. The accuracy, specificity, and sensitivity of all models are shown in Table 5.

**Table 5:** The performance of models in the training cohort and external validation cohort.

Cohort	Models	AUC	Accuracy	Specificity	Sensitivity
Training cohort	Laboratory parameter model	0.706 (95% CI: 0.607–0.804)	0.719	0.884	0.500
	Clinical model	0.903 (95% CI: 0.845–0.961)	0.860	0.899	0.808
	Imaging model	0.978 (95% CI: 0.957–0.997)	0.926	0.913	0.942
	Nomogram (clinical + imaiging)	0.982 (95% CI: 0.959–1.000)	0.942	0.942	0.942
External validation cohort	Laboratory parameter model	0.647 (95% CI: 0.477–0.812)	0.698	0.750	0.632
	Clinical model	0.761 (95% CI: 0.608–0.914)	0.791	0.917	0.632
	Imaging model	0.897 (95% CI: 0.834–0.995)	0.860	0.875	0.842
	Nomogram (clinical + imaiging)	0.921 (95% CI: 0.838–1.000)	0.860	0.792	0.945

AUC, area under the curve; CI, confidence interval.

## 4. Discussion

With the increasing incidence and prevalence of PIL and CD year by year, the differential diagnosis of PIL and CD has been widely and continuously concerned. In recent years, endoscopic biopsy and imaging have provided great assistance in the diagnosis of the disease. However, when the lesion is small in size or deep in location, endoscopy cannot accurately obtain a specimen suitable for diagnosis (3). In addition, endoscopic biopsy being an invasive test, the increased depth of sampling carries the risk of perforation because of the thin wall of the small intestine. Imaging evaluation plays an important role in the diagnosis of PIL and CD. In general, the thickening of the intestinal wall of about 2 cm contributes to the diagnosis of lymphoma (14). However, our study showed that approximately 62% of PIL patients did not achieve a thickening degree of 2 cm, with the minimum being only 10.18 mm. Additionally, PIL with different pathological types may exhibit imaging features similar to those of CD thereby influencing the radiologist’s judgment. This study aimed to develop a non-invasive model to provide valuable assistance for the differentiation of PIL and CD.

Many previous studies have attempted to differentiate PIL from CD and have made good progress. Zhang et al. (3) developed a highly sensitive and specific model for discriminating CD from PIL by collecting laboratory indices, clinical parameters, endoscopic features, and imaging features with an area under the ROC curve of 0.989. Recently, Yang et al. (15) established a differential diagnosis scoring model for CD versus ulcerative primary intestinal lymphoma (UPIL) based on clinical symptoms, endoscopic and imaging features. The accuracy of the model was as high as 83.66%. Meanwhile, the scoring model also showed high performance in the internal validation set, with an area under the ROC curve of 0.901. However, previous studies have built only one model to discriminate PIL from CD. In addition, the lack of an external validation cohort and the single evaluation metric may not provide an adequate assessment of the robustness of the model.

In this study, we first developed a laboratory parameters model. The AUC of the laboratory parameters model was greater than 0.69 in both the training and external validation cohort, with specificity exceeding 0.75. However, although the model had a high specificity but a low sensitivity of 0.632. In addition, the DCA curve showed a low patient benefit. Then, we developed a clinical model based on demographic and clinical symptoms. The clinical model had a higher AUC, accuracy, and specificity than the laboratory parameters model, with a specificity of 0.917. However, the sensitivity of the clinical model is similar to that of the laboratory parameters model. After that, we built an imaging features model based on CT images with an AUC as high as 0.897 in the external validation cohort, and the accuracy, specificity, and sensitivity of the model were over 0.80. Finally, we combined the indicators with *p* < 0.05 in the three models to build a nomogram with simplified indicators and high diagnostic performance.

Since there were no *p* < 0.05 indicators in the laboratory parameters model, the nomogram was finally built based on clinical and imaging features. Nomogram had an AUC of 0.921 in the external validation cohort, with a sensitivity of over 0.90, and its accuracy and specificity are similar to those of the imaging features model. Unlike previous studies, in addition to using the AUC for each model evaluation, the DeLong test was also conducted in this study. The study showed that the nomogram not only has a higher AUC but also a higher diagnostic efficacy than both laboratory and clinical models (*p* < 0.05). In addition, although the nomogram had a slightly higher AUC than the imaging features model, the difference was not statistically significant (*p* > 0.05). This indicates that the discrimination efficiency of the imaging features model is not lower than that of the nomogram. However, the indicators of the imaging features model are complex and easily influenced by subjective factors. In contrast, the nomogram includes only five indicators, which are simple and easily accessible. Therefore, we believe that the nomogram can simplify the indicators and improve diagnostic efficiency while maintaining high diagnostic efficacy.

Compared with Zhang’s study, our nomogram did not incorporate endoscopic features, which may explain the slightly lower AUC of the model. However, the AUC of our nomogram was slightly higher than Yang’s study and had higher sensitivity and accuracy. More importantly, compared to Zhang’s differential model that incorporated nineteen variables, we used five features to obtain a model with similar efficacy, simplifying the process of differential diagnosis. In addition, unlike the calibration curves plotted by Yang et al., we used the Bootstrap method to plot calibration curves after 1,000 sampling of data from Center 1, and the results showed that the calibrated nomogram still had good consistency and stability. Also, in this study, the patient benefit of each model was examined by DCA curves, and the study showed that the nomogram had the highest net benefit of all models in most of the threshold ranges in both cohorts. Finally, our study added data from Center 2 as an external validation cohort to comprehensively evaluate the performance of the nomogram. In the external validation cohort, the nomogram had a sensitivity of 0.945, accuracy and specificity of 0.86 and 0.79, respectively, and an area under the ROC curve of 0.921. The nomogram in this study contains simple and easily accessible indicators and features that may provide greater diagnostic value for rassroots hospital.

In clinical practice, PIL and CD may lead to misjudgment by clinicians due to similar clinical symptoms and endoscopic presentation. Kammal et al. (16) published a case report of misdiagnosis of NK/T-cell lymphoma as Crohn’s disease in 2019. However, the treatment of PIL is quite different from CD. PIL advocates for radiotherapy while CD mostly opts for drug therapy. Misdiagnosis or delayed diagnosis of the disease may lead to complications that may affect the patient’s prognosis. Therefore, in practice, when non-specific clinical symptoms and pathologic examination cannot accurately distinguish PIL from CD, the nomogram can provide physicians with new diagnostic ideas based on clinical symptoms and imaging signs to better distinguish PIL from CD. Especially in primary hospitals where pathology and diagnostic imaging capabilities are inadequate, a simple nomogram can be used to assist in the diagnosis of PIL when it is difficult to differentiate it from CD. Finally, we can collect feedback on the diagnostic accuracy and more valuable distinguishing features of each hospital to continuously optimize the nomogram. A better model can be built based on a larger sample size.

This study has some limitations. Firstly, the sample size of the study was small, which may be related to the low prevalence of PIL and CD. The nomogram we developed needs further validation and modification. Secondly, this is a retrospective study, and the missing data and the different scanning machines maybe affect the accuracy of the model. In addition, our collection of PIL patients included multiple pathological types. In the future, we look forward to exploring the differences between different subtypes of PIL and CD.

In conclusion, we explored a nomogram based on clinical data and CT images to easily and effectively distinguish PIL from CD. It is expected to provide valuable clues for clinical diagnosis and treatment.

## Data availability statement

The original contributions presented in the study are included in the article/Supplementary material, further inquiries can be directed to the corresponding author.

## Ethics statement

The studies involving humans were approved by The Ethics Committee of Shandong Provincial Hospital Affiliated to Shandong First Medical University, China. The studies were conducted in accordance with the local legislation and institutional requirements. Written informed consent for participation was not required from the participants or the participants’ legal guardians/next of kin in accordance with the national legislation and institutional requirements.

## Author contributions

HW was the guarantor. MX was involved in statistical analysis, data collection, and entry. MX, JT, HL, CQ, YM, and HW were involved in study conduct, manuscript draft, and revision. All authors contributed to the article and approved the submitted version.

## Funding

This study was supported by Key R&D Program of Shandong Province, China (2021SFGC0104).

## Conflict of interest

The authors declare that the research was conducted in the absence of any commercial or financial relationships that could be construed as a potential conflict of interest.

## Publisher’s note

All claims expressed in this article are solely those of the authors and do not necessarily represent those of their affiliated organizations, or those of the publisher, the editors and the reviewers. Any product that may be evaluated in this article, or claim that may be made by its manufacturer, is not guaranteed or endorsed by the publisher.
