# Supplementary material for: Nomogram based on computed tomography images and clinical data for distinguishing between primary intestinal lymphoma and Crohn’s disease: a retrospective multicenter study
Source: Front Med (Lausanne). 2023 Aug 17;10:1246861. doi: 10.3389/fmed.2023.1246861 (PMC10469891; doi:10.3389/fmed.2023.1246861)
Supplement: Supplementary file 1 [file Table_1.DOCX]

Supplementary Material

Nomogram based on CT images and clinical data for distinguishing between primary intestinal lymphoma and Crohn's disease: a retrospective multicenter study

**Mengjun Xiao, Jiahe Tan, Haiou Li, Chenyang Qiu, Yinchao Ma, Haiyan Wang***

*** Correspondence:**

Haiyan Wang

# [whyott@163.com](mailto:whyott@163.com)

# Supplementary Tables

| Supplementary Table 1. CT scan protocols | | | | |
| --- | --- | --- | --- | --- |
| Modality | CT | | | |
| Scanner model | SOMATOM Force | SOMATOM Definition Flash | Aquilion ONE | Discovety 750 |
| Manufacturer | Siemens | Siemens | Toshiba | General electric |
| Tube voltage(kV) | 120 | 120 | 120 | 120 |
| Tube current | 113 | 190 | 104 | 180 |
| Matrix | 512×512 | 512×512 | 512×512 | 512×512 |
| Slice thickness(mm) | 5 | 5 | 5 | 5 |
| Exposure time(ms) | 500 | 500 | 500 | 600 |
| Abbreviation: kV, kilovoltr; mm, millimeter; ms, millisecond. | | | | |

**
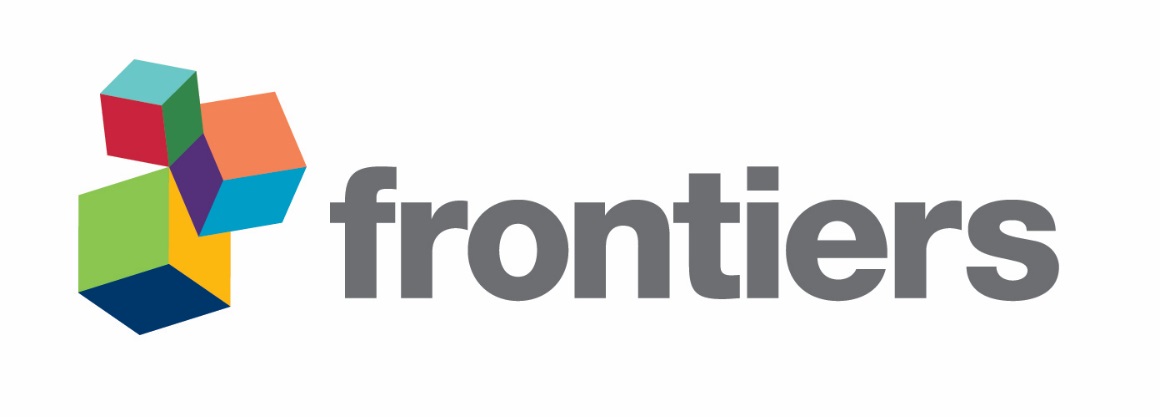
**
